# Supplementary material for: Silk Fibroin Coated Magnesium Oxide Nanospheres: A Biocompatible and Biodegradable Tool for Noninvasive Bioimaging Applications
Source: Nanomaterials (Basel). 2021 Mar 10;11(3):695. doi: 10.3390/nano11030695 (PMC7998877; doi:10.3390/nano11030695)
Supplement: Supplementary file 1 [file nanomaterials-11-00695-s001.pdf]

Supporting Information

Silk fibroin coated magnesium oxide nanospheres: A Biocompatible and biodegradable tool for non-invasive bioimaging applications

Jitao Li[1,2], Asma Khalid[2,3*], Rajni Verma[2], Amanda N. Abraham[3], Farah Qazi[2], Xiuxiu Dong[4], Gaofeng Liang[5] and Snjezana Tomljenovic-Hanic[2]

[1] School of Physics and Telecommunications Engineering, Zhoukou Normal University, Zhoukou 466001, China

[2]School of Physics, The University of Melbourne, Parkville 3010, Australia

[3] ARC Centre of Excellence for Nanoscale BioPhotonics, School of Science, RMIT University, Melbourne, Victoria 3001, Australia

[4] State Key Laboratory of Bioelectronics, School of Biological Science and Medical Engineering, Southeast University, Nanjing 210096, China

[5] Medical College, Henan University of Science and Technology, Luoyang 471023, Henan, China

*asma.khalid@rmit.edu.au

Section S1: Dynamic light scattering

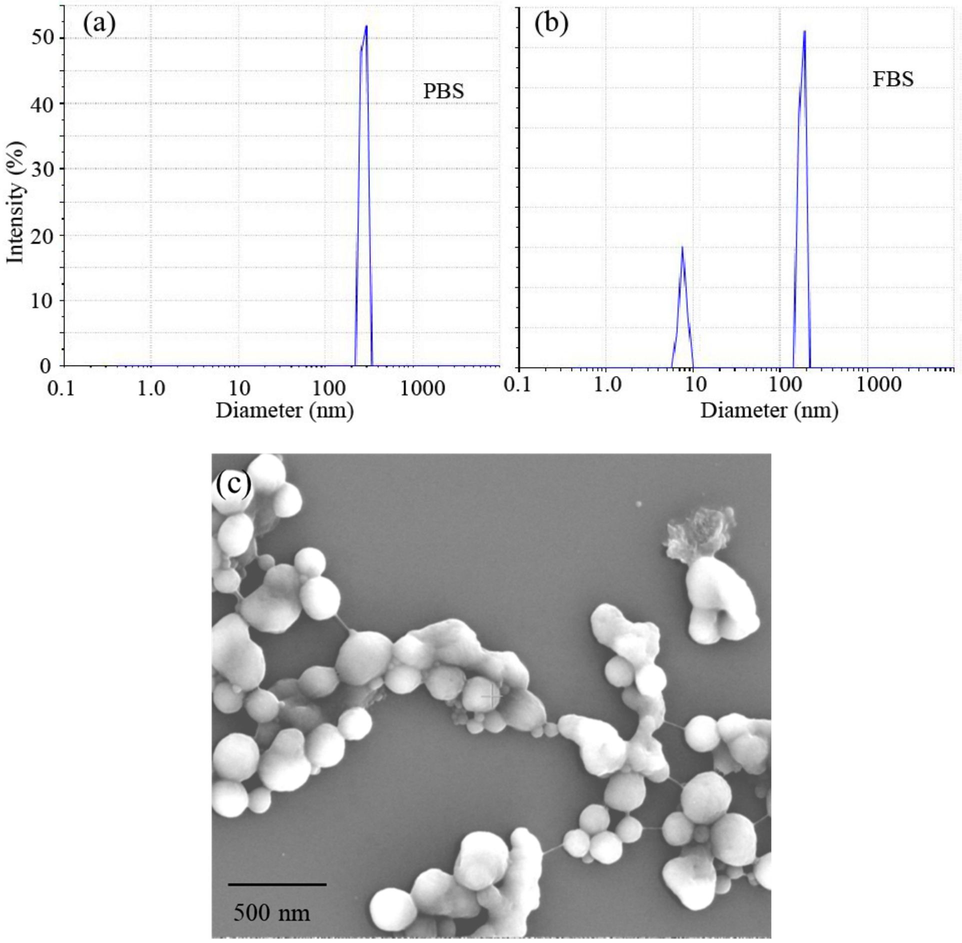

Figure S1: Hydrodynamic size distribution of size-reduced MgO-SF spheres in (a) PBS and (b) 10%FBS in PBS. The smaller peak in (b) around 10 nm is due to the scattering from the FBS protein aggregates (c) FESEM micrograph of size-reduced spheres at 40× magnification.

Section S2: Photoluminescence of MgO

(i) Color centres responsible for broad spectral data: To find the origin of the broad PL of MgO, we also recorded the PL spectrum at a low temperature under 532 nm excitation. excitation. The photoluminescence (PL) for MgO NPs, recorded at low temperature of -195 °C (under 532 nm excitation), shows a broad spectrum in Fig S1. The data again revealed a similar response (Figure 2b) with no characteristic peaks and a maximum intensity around 540 nm. This kind of fluorescence is attributed to F centre. Extensive defect PL studies on different forms of MgO have been performed for decades, where the most prominently discussed point defects are the anionic F and F+ centres, which contain an oxygen vacancy. The F centre emission is thus visible at around 530 nm or 540nm (as shown near 530 nm in Fig S2b) due to a broad PL. The F+ centre has been found to emit at much lower wavelengths of around 390 nm[1-3].

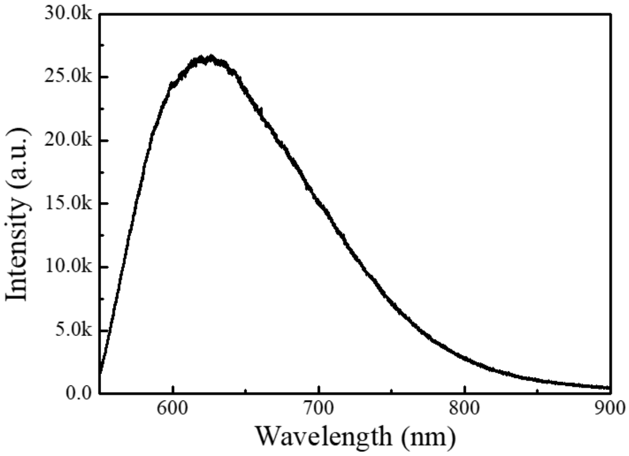

Figure S2: Emission spectra of MgO NPs recorded at low temperature (-195 °C) at excitation wavelength of 532 nm.

(ii) Color centres responsible for NIR emission: According to literature[4], the room temperature NIR PL spectra consist of the zero-phonon line (ZPL) and sideband luminescence observed from the chromium Cr^{3+} and V^{2+} ions. The broad fluorescence with a maximum at about 800 nm is assigned to the $^4T_2$-$^4A_2$ transition from tetragonal site of Cr^{3+} embedded in the MgO matrix. The ZPL assigned as the magnetic dipole (no-phonon) ^2E-$^4A_2$ transition appear around 688 and 700 nm arising from Cr^{3+} in cubic (R-line) and non-cubic (N-line), respectively[5-6]. They are observed only during the 532 nm excitation. Similar behavior exhibits second narrow band peaking at 721 nm is associated with vibronic side bands of the N-line for Cr^{3+} ions[7]. Moreover, the Vanadium V^{2+} ions are reported to fluoresce with peaks above 800 nm[6]. According to literature within 800-900 nm, there are 4 well separated peaks associated to V^{2+} ions that appear around 837 nm, 852 nm, 870 nm, 891 nm[6]

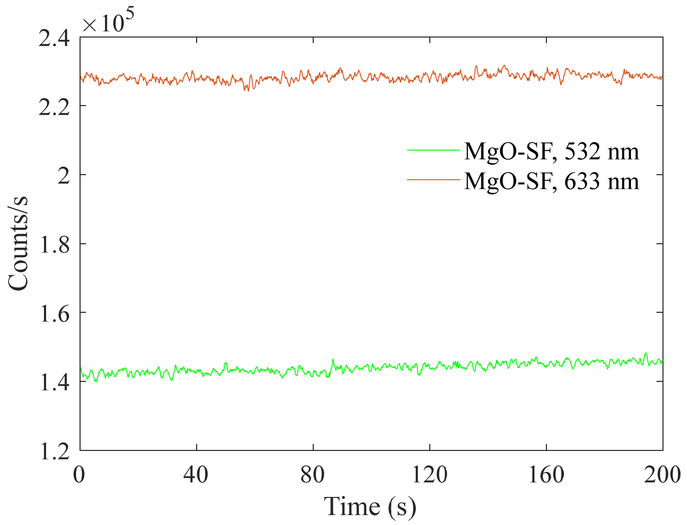

Figure S3: Emission counts of two representative photostable centres with emission in NIR regions, found in MgO NPs tracked for excitation wavelength of 532 (green trace) and 633 nm (red trace).

Hence for the low occurring emission centres of Fig 2 c,d (main manuscript), the spectral features below 800 nm are contributed by the Cr^{3+} ions. Whereas peaks visible on the right side of the broad band (above 800 nm) in Fig 2c are known to correspond to the V^{2+} ions. The intensity of the sideband lines at 837 nm and 852 nm are most likely the result of a phonon assisted transition. The line at 870 nm has been attributed to a magnetic dipole allowed and described as the ZPL of the ^2E_g-$^4A_{2g}$ transition in the MgO with V^{2+} system (S line). The peak at 891 nm have been reported to be the sidebands of the R line and assigned to an electric dipole allowed transition[6]. Hence, fabrication methods induce different types of vacancies, as discussed in our previous work [8]. Moreover the highly photostable count traces for two of these centres are shown in Fig S3.

Section S3: Cell viability

In addition to this, time and concentration dependent cell viability were assessed on HaCaT and U87MG cells as shown in Figure S4. It should be noted that cell viability is considered equal to the control/untreated (0 µg/mL) if % viability falls between 80%-120%[9]. Therefore, both the MgO-SF spheres (Figure S6 a,b) and the MgO NPs (Figure S6 C&D) show no biologically significant effect on cell viability on either cell line, up to 48 h. This is despite the statistically significant differences indicated by the asterisks in the figures. The MgO NPs cell viability results were similarly reported in our previously published work on HaCaT cells[10] and the U87MG results are in agreement with those observed by Lai *et al.*[11] After 72 h, ~25% cell death was observed at concentrations of 12.5 µg/mL and above with the U87MG cells, with both NPs. On the other hand, there was no significant effect observed on the HaCaT cells. This difference between the two cell lines may be attributed to the higher uptake of both NPs in the U87MG cells, in comparison to the HaCaT cells. There is also no significant difference between the MgO NPs and MgO-SF spheres data within each cell line, which suggests there is no further effects on the cell viability due to the presence of silk on the MgO NPs.

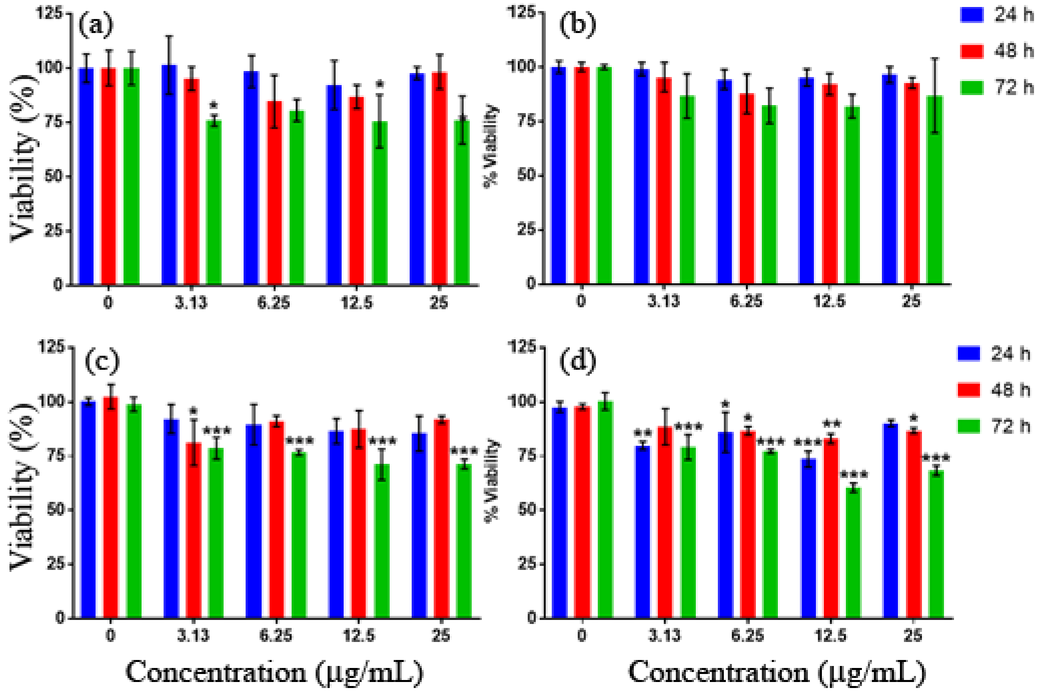

Figure S4: (a,b) Concentrations of MgO-SF nanospheres and (c,d) MgO NPs cultured in HaCaT cells and U87MG cells. $^*p<0.05$, $^{**}p<0.01$ and $^{***}p<0.001$ in comparison to the untreated cells (0 µg/mL) at the respective time points.

Section S4: Wide-field cell imaging for (48 h NP- incubation)

(i) HaCaT cells

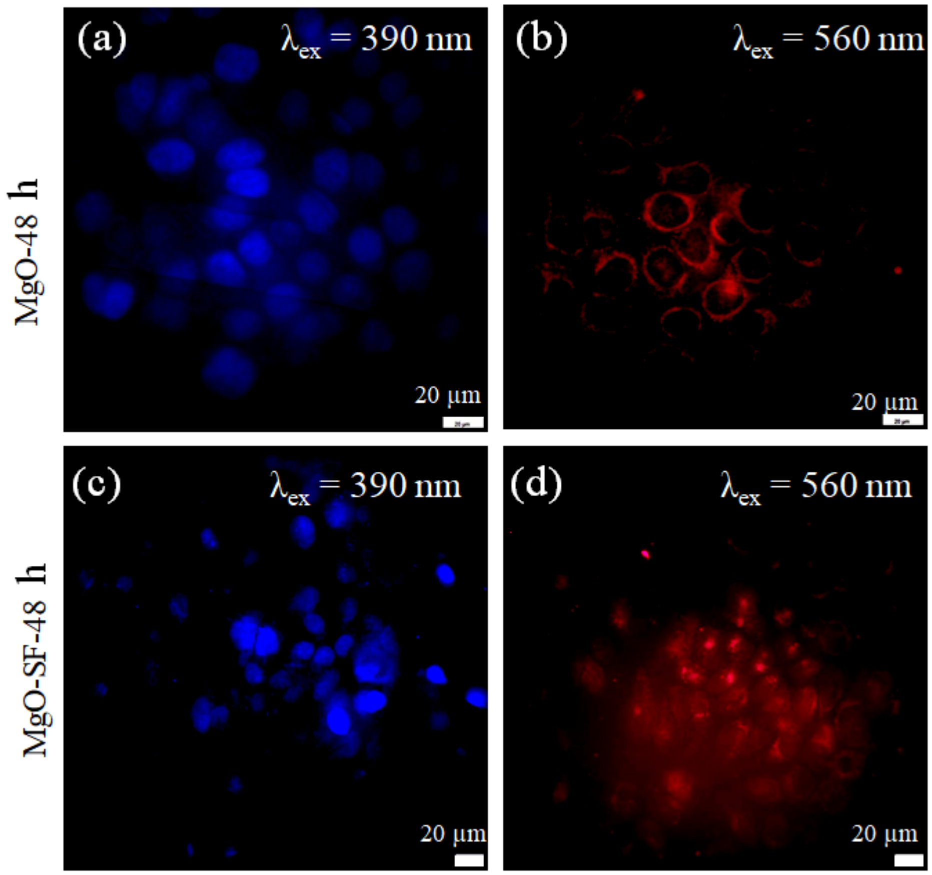

Figure S5: Confocal microscope images of HaCaT cells cultured with 5 µg/ml of (a,b) MgO-silk NPs after 48 hours of incubation under 390 and 560 nm excitation. The bars in each picture represents 20 µm. Even after 48 h, MgO-SF particles fluorescing brightly inside the cells.

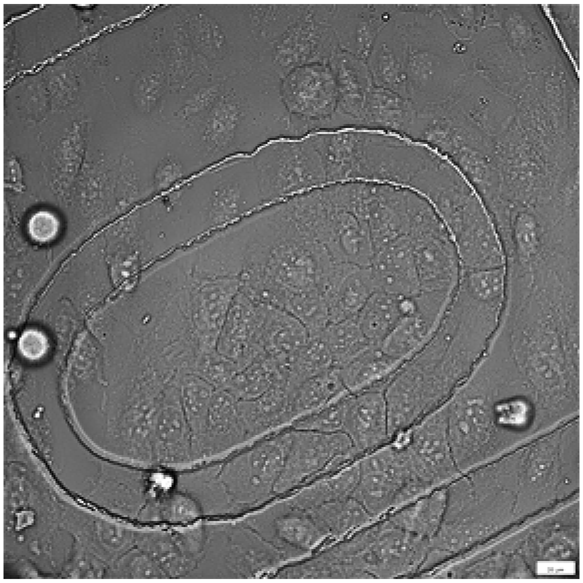

Figure S5ii: Bright field (white light transmission) image of HaCaT cells cultured with 5 µg/ml of MgO-silk spheres after 48 hours of incubation. The scale bar represents 20 µm.

(ii) U87MG cells

Fluorescence images of MgO-SF cultured brain cancer cells excited with 390, 485nm and 560 nm wavelengths, as shown in Fig S6. Images clearly show higher internalization and brighter fluorescence for the MgO-SF hybrid spheres within cells, indicated by distinct point fluorescence in Fig S6(b) compared to MgO alone in Figure S6 (a). Moreover green fluorescence from MgO-SF at excitation of 485nm is shown in Fig S6(c), as silk fibroin has a fluorescence maximum under blue (485 nm) excitation, while 560 nm excitation causes fluoresces of the MgO NPs (Fig S6(b)) in the hybrid MgO-SF spheres.

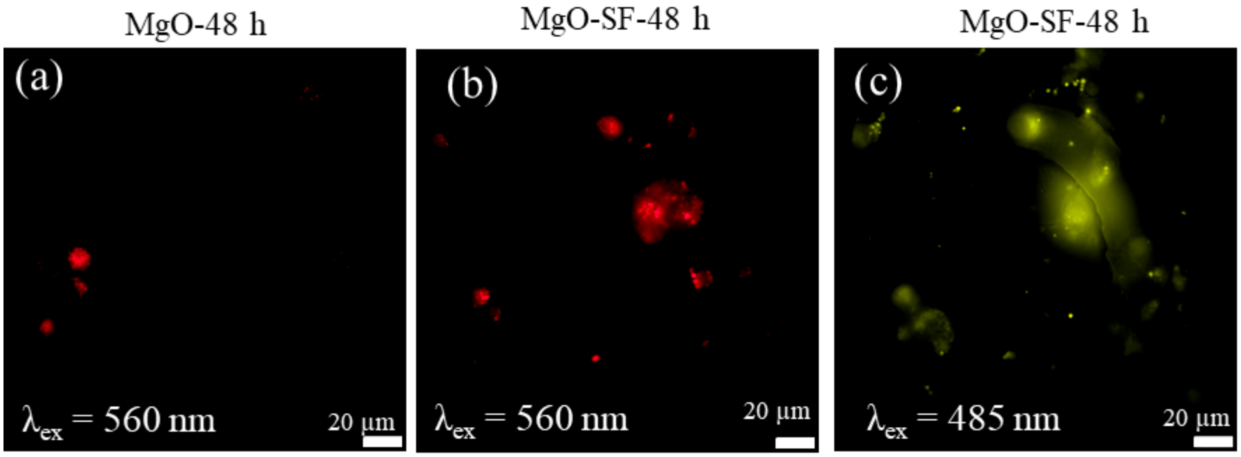

Figure S6: Wide field fluorescence of U87MG cells cultured with 5 µg/ml of (a) MgO NPs, (b-c) MgO-SF NPs after 48 h of incubation upon excitation with (a,b) 560 nm and (c) 485 nm. The bars in each picture represents 20 µm.

(iii) MCF7 cells

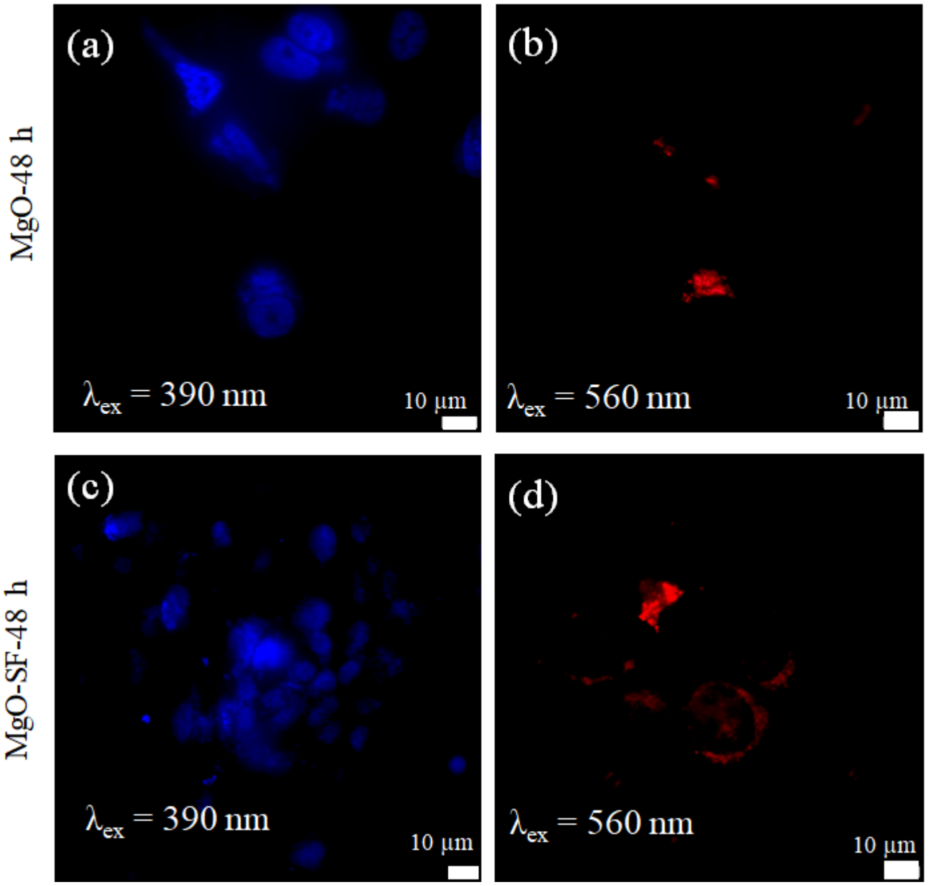

Figure S7: Wide field fluorescence of MCF7 cells cultured with 5 µg/ml of (a,b) MgO NPs, (c,d) MgO-SF NPs imaged after 48 h of incubation upon excitation with 390 nm and 560 nm. The bars in each picture represents 10 µm.

References

(1) Feldbach, E.; Jaaniso, R.; Kodu, M.; Denks, V. P.; Kasikov, A.; Liblik, P.; Maaroos, A.; Mändar, H.; Kirm, M. Luminescence characterization of ultrathin MgO films of high crystallinity prepared by pulsed laser deposition. *Journal of Materials Science: Materials in Electronics* **2009,** *20* (1), 321-325, DOI: 10.1007/s10854-008-9599-z.
(2) Rosenblatt, G. H.; Rowe, M. W.; Williams, G. P.; Williams, R. T.; Chen, Y. Luminescence of F and ${F}^{+}$ centers in magnesium oxide. *Physical Review B* **1989,** *39* (14), 10309-10318, DOI: 10.1103/PhysRevB.39.10309.
(3) Summers, G. P.; Wilson, T. M.; Jeffries, B. T.; Tohver, H. T.; Chen, Y.; Abraham, M. M. Luminescence from oxygen vacancies in MgO crystals thermochemically reduced at high temperatures. *Physical Review B* **1983,** *27* (2), 1283-1291, DOI: 10.1103/PhysRevB.27.1283.
(4) Feldbach, E.; Kirm, M.; Kozlova, J.; Maaroos, A.; Mändar, H.; Saar, R.; Sammelselg, V. Luminescence spectroscopy of nanocrystalline MgO. *physica status solidi c* **2011,** *8* (9), 2669-2672, DOI: 10.1002/pssc.201084091.
(5) Cossolino, L. C.; Zanatta, A. R. Influence of chromium concentration on the optical–electronic properties of ruby microstructures. *Journal of Physics D: Applied Physics* **2009,** *43* (1), 015302, DOI: 10.1088/0022-3727/43/1/015302.
(6) Prucnal, S.; Shalimov, A.; Ozerov, M.; Potzger, K.; Skorupa, W. Magnetic and optical properties of virgin arc furnace grown MgO crystals. *Journal of Crystal Growth* **2012,** *339* (1), 70-74, DOI: https://doi.org/10.1016/j.jcrysgro.2011.11.067.
(7) Larkin, J. P.; Imbusch, G. F.; Dravnieks, F. Optical Absorption in MgO: ${\mathrm{Cr}}^{3+}$. *Physical Review B* **1973,** *7* (1), 495-500, DOI: 10.1103/PhysRevB.7.495.
(8) Chaudhri, M. M.; Sands, H. S. Photoluminescence from indented MgO crystals using a near ultraviolet/visible Raman microscope. *Journal of Applied Physics* **1997,** *82*, 785, DOI: 10.1063/1.365773.

(9) Hemelaar, S. R.; Saspaanithy, B.; L'Hommelet, S. R. M.; Perona Martinez, F. P.; van der Laan, K. J.; Schirhagl, R. The Response of HeLa Cells to Fluorescent NanoDiamond Uptake. *Sensors (Basel)* **2018,** *18* (2), 355, DOI: 10.3390/s18020355.
(10) Khalid, A.; Norello, R.; N. Abraham, A.; Tetienne, J.-P.; J. Karle, T.; W. C. Lui, E.; Xia, K.; A. Tran, P.; J. O'Connor, A.; G. Mann, B.; de Boer, R.; He, Y.; Man Ching Ng, A.; B. Djurisic, A.; Shukla, R.; Tomljenovic-Hanic, S. Biocompatible and Biodegradable Magnesium Oxide Nanoparticles with In Vitro Photostable Near-Infrared Emission: Short-Term Fluorescent Markers. *Nanomaterials* **2019,** *9* (10), 1360.
(11) Lai, J. C. K.; Lai, M. B.; Jandhyam, S.; Dukhande, V. V.; Bhushan, A.; Daniels, C. K.; Leung, S. W. Exposure to titanium dioxide and other metallic oxide nanoparticles induces cytotoxicity on human neural cells and fibroblasts. *Int J Nanomedicine* **2008,** *3* (4), 533-545, DOI: 10.2147/ijn.s3234.
